# Engineering *ε*-polylysine-based photodynamic therapy agents with oxygen carrying and membrane-targeting capabilities for enhanced therapy under hypoxic conditions

**DOI:** 10.1039/d5ra05124j

**Published:** 2026-01-05

**Authors:** Lianjun Mou, Xiaoyan Lou, Chang Liu, Qian Yuan, Qingmeng Zhang, Xilei Xie, Xiaoyun Jiao, Heng Liu, Jian Zhang

**Affiliations:** a Department of Pathology, Hunan University of Medicine General Hospital Huaihua 418000 China; b College of Chemistry, Chemical Engineering and Materials Science, Shandong Normal University Jinan 250014 China zhangjian12b@mails.ucas.ac.cn; c Department of Orthopaedics, Qilu Hospital of Shandong University Jinan 250012 China zhangqingmeng@email.sdu.edu.cn; d Key Laboratory of Reproductive Health Diseases Research and Translation of Ministry of Education, The First Affiliated Hospital, Hainan Medical University Haikou 571199 China liuheng11b@muhn.edu.cn; e State Key Laboratory of Molecular Engineering of Polymers, Fudan University Shanghai 200438 China

## Abstract

Photodynamic therapy (PDT) offers exceptional spatial and temporal precision, minimal invasiveness, and negligible side effects. However, the hypoxic microenvironment of tumors greatly limits the effectiveness of conventional photodynamic therapy (PDT) and severely reduces its therapeutic efficiency. Here, a simple and versatile method for the preparation of PDT nanocomplexes based on *ε*-polylysine (PLY-1/2/3) was developed. By covalently combining a photosensitiser 5,10,15,20-tetra (4-carboxyl phenyl) porphyrin (H_2_TCPP), a biotin-targeting moiety (d-Bio) and an O_2_-carrying agent, perfluorohexanoic acid (PF-HA), the nanocomplex achieved specific targeting of HeLa cell membranes and was capable of efficiently generating cytotoxic singlet oxygen (^1^O_2_) in both normoxic and hypoxic conditions. Furthermore, O_2_-loaded PLY-1 had enhanced PDT efficacy, and it also showed a higher degree of apoptosis than the non-O_2_-loaded one. The results provided a facile and general method for the design and preparation of PDT composite against hypoxia.

## Abstract 2

Photodynamic therapy (PDT) offers exceptional spatial and temporal precision, minimal invasiveness, and negligible side effects.

## Graphical abstract

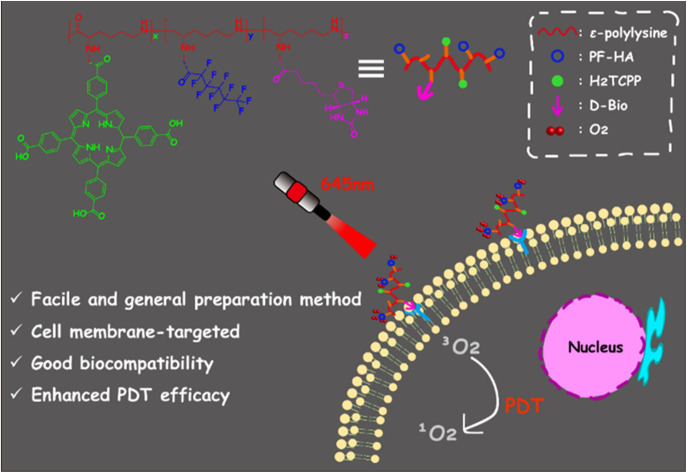

## Introduction

Since cancer endangers human life and health seriously, the research on cancer treatment is of great significance.^1^ Compared with traditional modalities such as surgical resection, radiotherapy, or chemotherapy, photodynamic therapy (PDT) has the advantages of super-spatial and temporal resolution, less invasiveness, and smaller side effects.^2,3^ Nevertheless, it is subject to photosensitizers, excitation light, and oxygen (O_2_), which should be requested simultaneously to achieve the killing of cancer cells.^4^ In order to improve the efficiency of PDT, a method widely adopted and proved to be effective is to integrate different mono-functional units to molecular skeletons or nano-carriers, and then construct multifunctional composites.^5–8^ Most of the reported composites used for PDT are prepared using mesoporous silica,^9–11^ metal–organic frameworks^12,13^^,^ and covalent organic frameworks,^14,15^ which are loaded and modified with several functional molecules, respectively. However, the preparation process is complicated with synthesized and modified steps as well as harsh conditions.^16,17^ Moreover, the prepared composites often are inclined to cause drug leakage and lead to biological toxicity, which limits further application.^18^ Therefore, it is necessary to develop a general method for facile preparation of PDT composites with stable structures and potent efficacy.^19^

As a typical feature of solid tumors, hypoxia results from the lack of oxygen supply, which is induced by the malignant proliferation of cancer cells and the hypoplasia of blood vessels. It is closely related to the occurrence of cancer development, and also limits the efficacy of conventional oxygen consumption therapies such as PDT and sonodynamic therapy.^20–22^ On the other hand, most of the existing commercial photosensitizers kill cancer cells by sensitizing O_2_ to produce toxic singlet oxygen (^1^O_2_). While the killing effect of these photosensitizers is remarkable only in the normoxic site of the tumor, it is forceless to the hypoxic site.^23^ Moreover, a large amount of O_2_ will be consumed in the course of PDT treatment, which further aggravates the degree of hypoxia and greatly impairs the efficacy of PDT.^24^ Therefore, it is critical to alleviate the hypoxic microenvironment and reduce the hypoxic resistance to enhance the efficacy of PDT.^25–28^

**Scheme 1:**
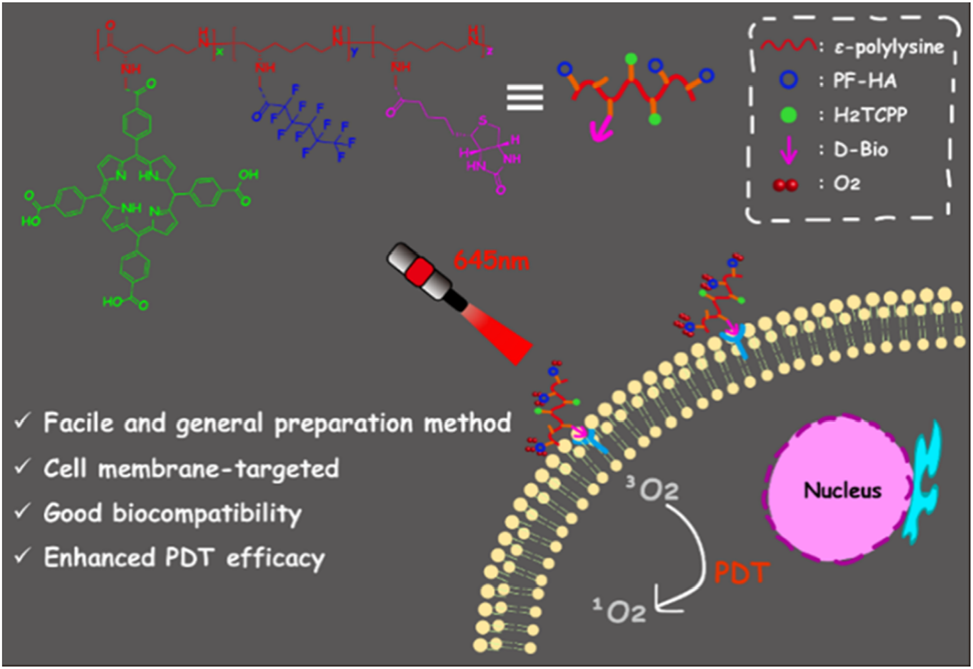
The graphic illustration of the preparation and application of PDT composite (PLY-1) based on *ε*-polylysine.

Herein, we have developed a simple and general method to prepare the PDT composites which loaded with different amounts of porphyrin (PLY-1/2/3). It was obtained by covalently grafting different functional units to *ε*-polylysine as a polypeptide skeleton. *ε*-polylysine has many advantages, such as cheap, good water solubility and thermal stability, favorable biocompatibility and degradability *in vivo*. Moreover, it has been used as a drug carrier.^29–31^ In the prepared PDT composites, the photosensitizer was 5,10,15,20-tetra (4-carboxyl phenyl) porphyrin (H_2_TCPP), which mediated the production of ^1^O_2_. Because of the specific binding ability to the biotin receptors on cancer cells,^32^ D-biotin (d-Bio) endowed the targeting feature to PLY-1; perfluorohexanoic acid (PF-HA) was served as an oxygen-carrying agent to supply O2 to relieve the hypoxic dilemma. It was revealed that PLY-1 could target the cell membranes of HeLa cells specifically and produce ^1^O_2_ to kill HeLa cells both in normoxia and hypoxia effectively (Scheme 1). Furthermore, O_2_-loaded PLY-1 had enhanced PDT efficacy. It also showed a higher degree of apoptosis than non-O_2_-loaded PLY-1. The results provided a facile and general method for the design and preparation of PDT composite against hypoxia.

## Experimental section

### Materials and methods

The materials, instruments, ^1^O_2_ generation, cell experiment, cytotoxicity assays, live and dead cell staining, and apoptosis test are listed in the SI.

### Synthesis of compound PLY-1

A mixture of 5,10,15,20-tetra (4-carboxyl phenyl) porphyrin (H_2_TCPP, 0.05 mmol, 40.0 mg), *N*-(3-dimethylaminopropyl)-*N*′-ethylcarbodiimide hydrochloride (EDC, 0.40 mmol, 76.7 mg), and *N*-hydroxysuccinimide (NHS, 0.40 mmol, 46.1 mg) was dissolved in DMF. The mixture was stirred at room temperature for 4 h in the dark. Subsequently, *ε*-polylysine (2.00 mmol, 316.0 mg) dissolved in DMF was added, and the mixture was stirred at 60 °C under argon protection for 4 h, and then the mixture was moved to room temperature for 20 h. At the same time, perfluorohexanoic acid (PF-HA, 0.20 mmol, 72.8 mg) and D-biotin (d-Bio, 0.01 mmol, 2.5 mg) were respectively activated by reacting with EDC and NHS in argon at 60 °C for 4 h, and stirring at room temperature for 20 h, then added to the above solution. After completion of the reaction, the reaction solution was dialyzed with a regenerated cellulose dialysis bag (MWCO: 1.0 KDa) (H_2_O :  EtOH = 3 : 7) for 72 h, and the dialysate was changed every 4 h. Finally, the PLY-1 was obtained by lyophilization.

## Results and discussion

**Fig. 1:**
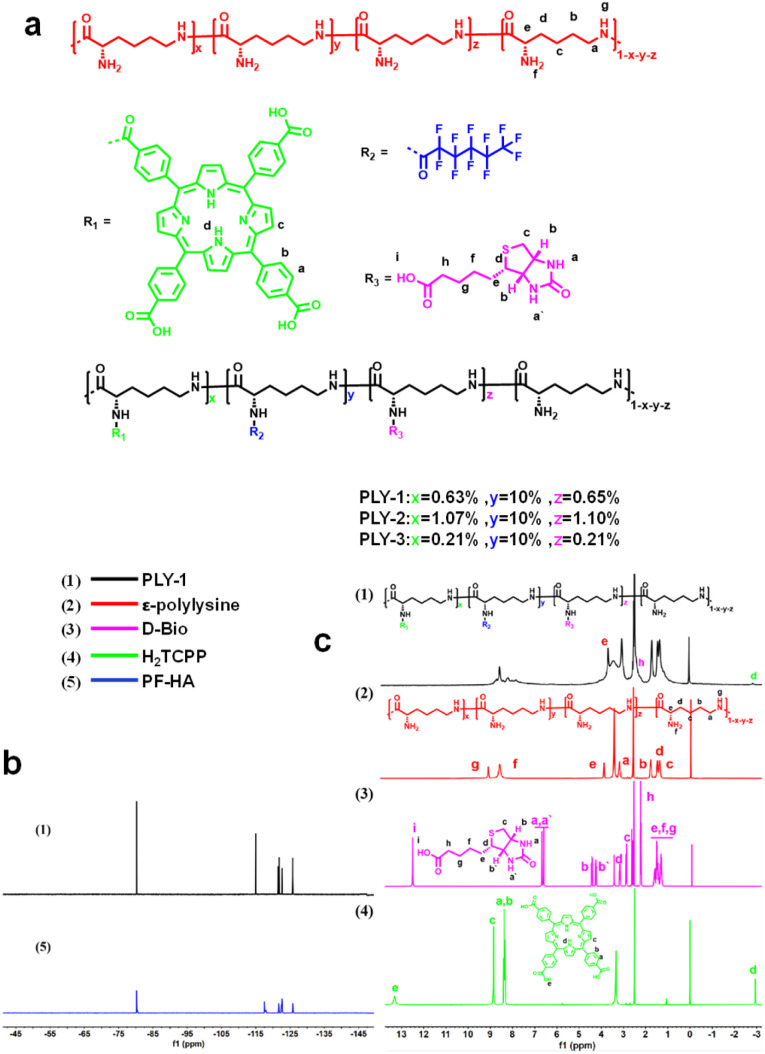
(a) The molecular structures of PLY-1/2/3; (b) ^19^F NMR spectra of (1) PLY-1 and (5) PF-HA in deuterated DMSO; (c) the ^1^H NMR spectra of (1) PLY-1; (2) *ε*-polylysine; (3) d-Bio and (4) H_2_TCPP in deuterated DMSO.

PLY-1/2/3, which can improve the PDT efficacy in hypoxia, has been designed and prepared by employing *ε*-polylysine as the skeleton (Fig. 1a). Specifically, H_2_TCPP, PF-HA, and d-Bio were covalently bonded to *ε*-polylysine by amide reaction that was mediated with *N*-(3-dimethylaminopropyl)-*N*′-ethylcarbodiimide hydrochloride and *N*-hydroxysuccinimide successively. After dialysis and lyophilization, PLY-1/2/3, which have different H_2_TCPP content, were obtained (Fig. S1–S7).

Firstly, the molecular structure of PLY-1 was characterized and confirmed by nuclear magnetic resonance (NMR) and Fourier transform infrared spectroscopy (FTIR). It could be seen from ^1^H NMR that the signal peaks at 3.76 ppm could be attributed to the methine hydrogen (–CO–CH (NH_2_)–) in the skeleton of *ε*-polylysine, respectively; The signal peaks at 8.50–8.58 ppm and 8.73 ppm were corresponded to H_2_TCPP; The signals at 2.59 ppm, 3.04–3.14 ppm were originated from d-Bio unit (Fig. 1c); ^19^F NMR showed that the signals in PLY-1 were consistent with the characteristic peaks in PF-HA (Fig. 1b). It was indicated that PF-HA, d-Bio, and H_2_TCPP were covalently bonded to the *ε*-polylysine skeleton, which was further supported by the results of FTIR (Fig. S8). The peaks at 1647 cm^−1^ and 1554 cm^−1^ in PLY-1 were attributed to the stretching vibration bands of the carboxyl groups in H_2_TCPP and ester carbonyl groups, respectively. The peaks at 3254 cm^−1^ (N–H stretching vibration), 1655 cm^−1^ (amide I band), and 1556 cm^−1^ (amide II band) corresponded to *ε*-polylysine. The peaks at 1000–1400 cm^−1^ were attributed to C–F bond in PF-HA, and the peak at 1364 cm^−1^ was the characteristic band of CF–CF_3_; The peak at 721 cm^−1^ was assigned to = C–H on the pyrrole ring. These results indicated that the PDT composites containing multifunctional units could be obtained by simple condensation and lyophilization.

**Fig. 2:**
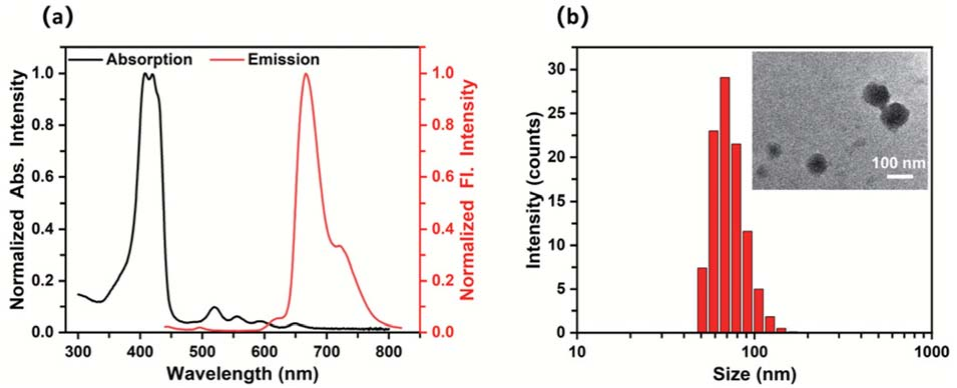
Photophysical properties of PLY-1. (a) The normalized absorption (black line) and emission (red line) spectra of PLY-1 (10.0 µg mL^−1^); (b) DLS and TEM (inset) results of PLY-1 (10.0 µg mL^−1^).

The absorption and emission spectra of PLY-1/2/3 were then collected, respectively (Fig. S9). While the H_2_TCPP content was increased from 0.21% (PLY-3) to 0.63% (PLY-1), both the absorption and emission intensity enhanced significantly. However, it weakened with the further increase of H_2_TCPP content from 0.63% (PLY-1) to 1.07% (PLY-2). It indicated that the observed PLY-1/2/3 might self-assemble in water, and the H_2_TCPP content of 1.07% (PLY-2) would result in the notorious aggregation-induced quenching (ACQ). Therefore, PLY-1, showing the best photophysical properties in the prepared PLY-1/2/3, was selected to carry out the subsequent experiment. The typical absorption and emission peaks attributed to H_2_TCPP could be observed clearly in Fig. 2a. Moreover, the particle diameter of PLY-1 was determined as ∼100 nm by dynamic light scattering (DLS) and transmission electron microscopy (TEM), which corroborated the self-assembly behavior of PLY-1 in water (Fig. 2b).

**Fig. 3:**
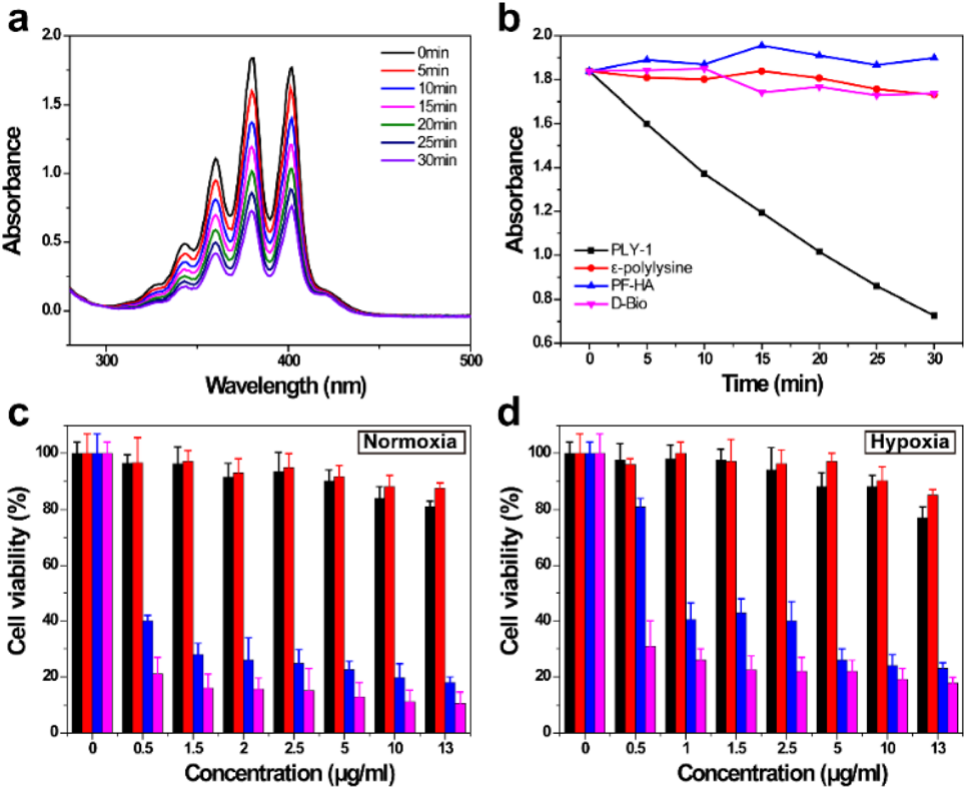
(a) Degradation curves of ABDA by PLY-1; (b) evolution curves of ABDA by PLY-1, *ε*-polylysine, PF-HA and d-Bio respectively; MTT assay of HeLa cells incubated with different concentrations of PLY-1 in normoxia (c) and hypoxia (d) respectively, in which different color represent different group, black: PLY-1; red: PLY-1 + O_2_; blue: PLY-1 + light; purple: PLY-1 + O_2_ + light; (light: 645 nm, 210 mW cm^−2^, 5 min).

Next, the photosensitive property of PLY-1 was verified with ABDA. The absorbance of ABDA decreased continuously with the illumination of 645 nm (Fig. 3a). At the same time, other functional units in PLY-1, including *ε*-polylysine, PF-HA, and d-Bio, hardly degraded ABDA under the same physiological conditions (Fig. 3b and S10), indicating that PLY-1 can produce ^1^O_2_ by H_2_TCPP with the irradiation of light at 645 nm.

**Fig. 4:**
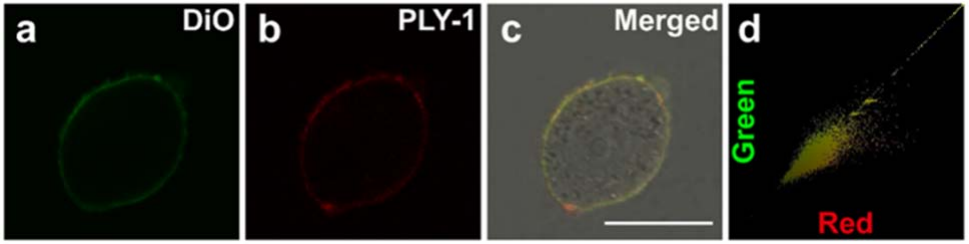
CLSM images of HeLa cells co-incubated with (a) Dio (5.0 µM, *λ*_ex_/*λ*_em_ = 488 nm/480–530 nm), and (b) PLY-1 (10.0 µg mL^−1^, *λ*_ex_/*λ*_em_ = 405 nm/600–750 nm); (c) merged image; (d) intensity correlation plot of Dio and PLY-1. Scale bar: 25 µm.

Since biotin receptors are overexpressed on the cell membrane of cancer cells, d-Bio was grafted to PLY-1 as the targeting group.^33^ We then examined the uptake ability of HeLa cells to PLY-1. It showed that the accumulation of PLY-1 on HeLa cell membranes gradually increased with the extension of incubation time, and the plateau was reached in 30 min (Fig. S11). Furthermore, the co-localization assay was also carried out with PLY-1 and DiO (a commercial cell membrane-tracking dye). It revealed that PLY-1 had excellent cell membrane targeting ability, and the co-localization coefficient was as high as 0.95 (Fig. 4).

Subsequently, the MTT assay was performed to evaluate the killing ability of PLY-1 against HeLa cells in normoxia and hypoxia, respectively (Fig. 3c and d). It exhibited insignificant dark toxicity (the cell survival rate was above 80%) to HeLa cells both in normoxia and hypoxia when the concentration of PLY-1 was 13.0 µg mL^−1^, which also meant that PLY-1 had good biocompatibility. In contrast, the viability of HeLa cells in normoxia was reduced to lower than 40% when 645 nm irradiation (5 min, 210 mW cm^−2^) was employed, and this was further decreased (lower than 20%) after PLY-1 was loaded with O_2_. PLY-1 also displayed similar phototoxicity to HeLa cells in hypoxia. Furthermore, PLY-1 loaded with O_2_ showed stronger lethality to HeLa cells than that without O_2_ loading. 2,7-Dichlorofluorescein diacetate (DCFH-DA) was then used to verify the ability of PLY-1 to generate ^1^O_2_ in HeLa cells. Compared with the blank group and the control groups, only the HeLa cells in the PLY-1 + light group generated bright green fluorescence (Fig. S12), which validated that PLY-1 could produce toxic ^1^O_2_ with the excitation of 645 nm to kill HeLa cells. To clarify whether this enhancement originates from hypoxia alleviation rather than intrinsic photophysical changes, HIF-1α expression was quantified under hypoxic conditions. O_2_-loaded PLY-1 significantly downregulated HIF-1α regardless of light irradiation, indicating effective hypoxia relief (Fig. S13).

**Fig. 5:**
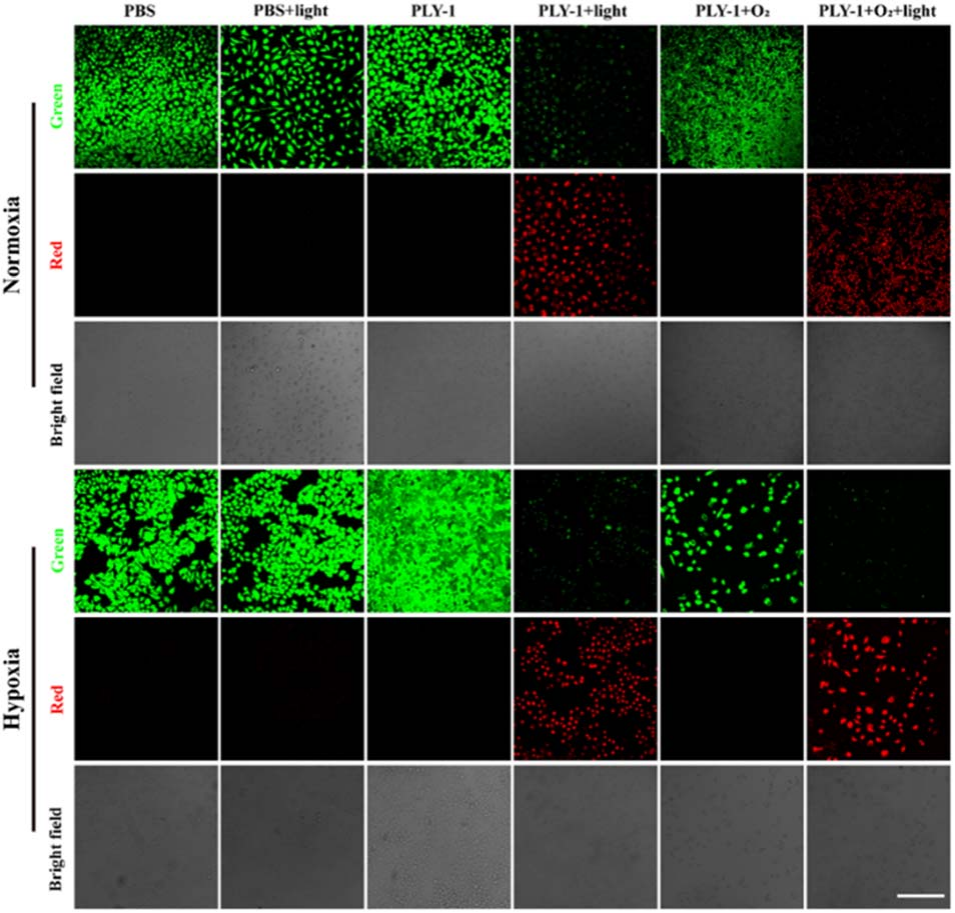
CLSM images of HeLa cells incubated with calcein-AM/PI after different treatments in normoxia and hypoxia, respectively. The treatments included PBS, PBS + light, PLY-1, PLY-1 + light, PLY-1 + O_2_ and PLY-1 + O_2_ + light groups (PLY-1: 10.0 µg mL^−1^, light: 645 nm, 210 mW cm^−2^, 5 min; Calcein-AM excitation source: 488 nm, collection channel: 500–550 nm, PI excitation source: 561 nm, collection channel: 580–650 nm, scale: 250 µm).

To further evaluate the applicability of PLY-1 as a PDT composite, we performed a live/dead cell staining assay (Fig. 5). While HeLa cells in PBS, PBS + light, PLY-1, and PLY-1 + O_2_ groups showed strong green fluorescence, the red channel was not significantly enhanced in normoxia and hypoxia. It was demonstrated that PLY-1 had good biosecurity, and the red light with 210 mW cm^−2^ did not damage HeLa cells. However, the PLY-1 + light and PLY-1 + O_2_ + light groups displayed distinct outputs. It was observed that the fluorescence of the green channel was decreased. Moreover, the weakness of the green channel in PLY-1 + O_2_ + light group was more significant than that of PLY-1 + light group. Meanwhile, the red fluorescence channel was significantly enhanced. This meant that ^1^O_2_ produced by PLY-1 under light mediated the death of HeLa cells. The ^1^O_2_-generated capacity of PLY-1 was further strengthened after O_2_-carrying, and the PDT efficacy was enhanced.

**Fig. 6:**
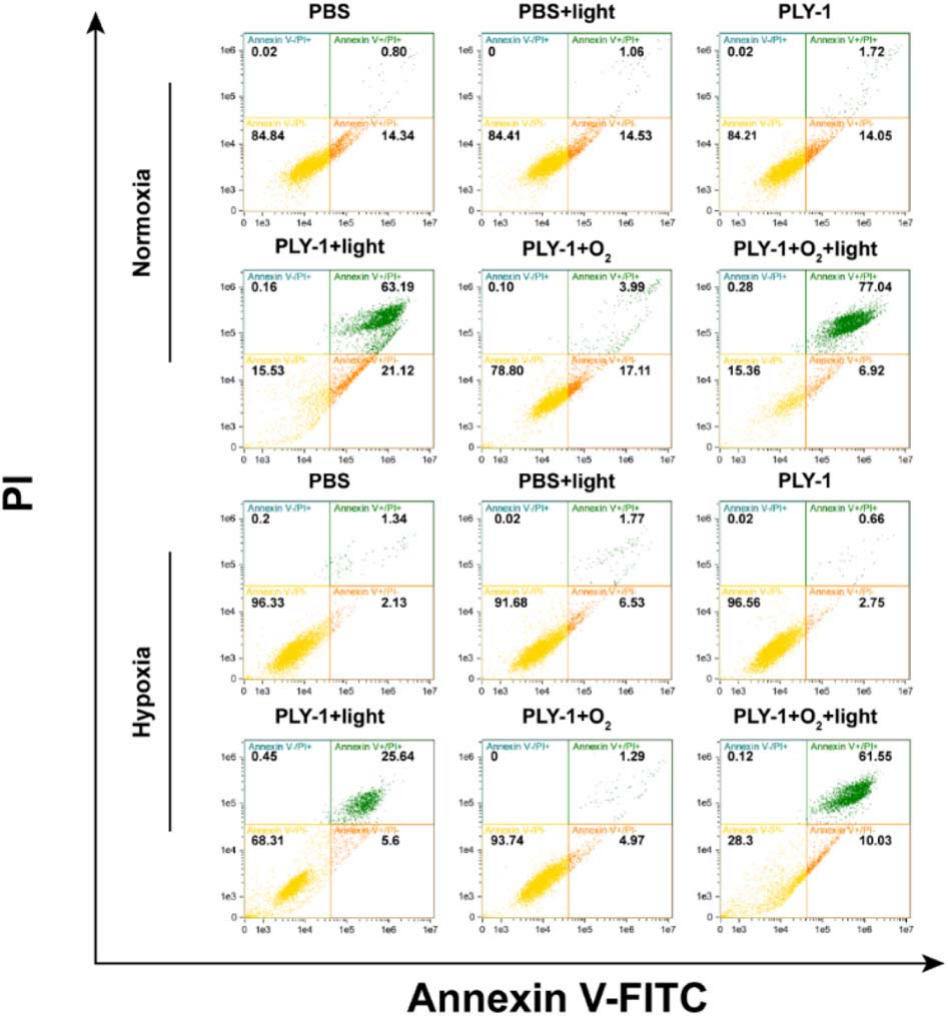
Flow cytometry apoptosis assay incubated with Annexin V-FITC and PI after different treatments in normoxia and hypoxia, respectively. The treatments included PBS, PBS + light, PLY-1, PLY-1 + light, PLY-1 + O_2_ and PLY-1 + O_2_ + light groups (PLY-1: 10.0 µg mL^−1^, light: 645 nm, 210 mW cm^−2^, 5 min; FITC excitation source: 488 nm, collection channel: 490–560 nm; PI excitation source: 561 nm, collection channel: 600–680 nm).

The apoptosis kits (YF488-Annexin V/PI) were implemented to further identify the different apoptosis stages in HeLa cells induced by PLY-1 (Fig. S14). During the course of apoptosis, phosphatidylserine would turn over to the outside of the cell membrane and bind with YF488-annexin V that could not penetrate the cell membrane, then emit green fluorescence, indicating the cells were in the early stage of apoptosis; when the cells were in late necrotic or apoptotic stage, propidium iodide (PI) would penetrate the damaged cell membrane rather than intact cell membrane, and emit red fluorescence. As can be seen in Fig. S14, there were no significant changes in the green fluorescence channel in PBS, PBS + light, PLY-1, and PLY-1 + O_2_ groups both in normoxia/hypoxia. However, the red fluorescence channel in the PLY-1 + light and PLY-1 + O_2_ + Light groups was lighted. In addition, the PLY-1 + O_2_ + light group showed stronger fluorescence than the PLY-1 + light group, which not only suggested that PLY-1 had very low dark toxicity, but also indicated that PLY-1 mediated the death of HeLa cells through late apoptosis or cell necrosis. Moreover, PLY-1 + O_2_ + light group displayed more HeLa cells in the late apoptotic or necrotic state compared to the PLY-1 + light group, showing a more significant PDT efficacy. This was consistent with the results of MTT and live/dead cell staining assay, and also verified by the flow cytometry (Fig. 6). Compared with PBS (0.80%), PBS + light (1.06%), PLY-1 (1.72%) and PLY-1 + O_2_ (3.99%) groups in normoxia, HeLa cells in PLY-1 + light and PLY-1 + O_2_ + light groups showed more apoptosis, and the proportion of cells in late apoptotic or necrotic stage increased to 63.19% and 77.04% respectively. A similar trend was also observed in hypoxia. The ratio was 1.34%, 1.77%, 0.66% and 1.29% in PBS, PBS + light, PLY-1, and PLY-1 + O_2_ groups, respectively, and increased to 25.64% and 61.55% in the PLY-1 + light and PLY-1 + O_2_ + light groups, indicating that O_2_-carried PLY-1 could induce more HeLa cells to be in the late apoptotic or necrotic stage, regardless of normoxia or hypoxia. Specifically, the enhancement of PDT efficacy in hypoxia was more significant than in normoxia when O_2_ was carried by PLY-1.

## Conclusions

In summary, a series of PDT composites (PLY-1/2/3) has been designed and prepared by employing *ε*-polylysine as the skeleton. H_2_TCPP served as the photosensitizer; d-Bio endowed the targeting ability to the PDT composites; PF-HA could carry O_2_ to ameliorate the hypoxic microenvironment. It was shown that PLY-1 had outstanding biocompatibility and could be targeted to the cell membranes of HeLa cells. Moreover, the PDT efficacy of PLY-1 was further enhanced both in normoxia and hypoxia when O_2_ was carried. It was highlighted that the design method proposed and adopted in this work is facile and general, which might be employed to deal with the current dilemma of complicated preparation steps for PDT composites. In addition, this work has confirmed the feasibility of this design method and provided a new view for the development of PDT composites. It was also expected to improve the efficacy of PDT against tumors in hypoxia.

## Conflicts of interest

The authors declare that they have no known competing financial interests or personal relationships that could have appeared to influence the work reported in this paper.

## Supplementary Material

RA-016-D5RA05124J-s001

## Data Availability

Data will be made available on request. Supplementary information (SI) is available. See DOI: https://doi.org/10.1039/d5ra05124j.
