# Supplementary material for: Engineering ε-polylysine-based photodynamic therapy agents with oxygen carrying and membrane-targeting capabilities for enhanced therapy under hypoxic conditions
Source: RSC Adv. 2026 Jan 5;16(2):1151–6. doi: 10.1039/d5ra05124j (PMC12766279; doi:10.1039/d5ra05124j)
Supplement: RA-016-D5RA05124J-s001 [file RA-016-D5RA05124J-s001.pdf]

Supplementary Information (SI) for RSC Advances.
This journal is © The Royal Society of Chemistry 2025

Supporting Information

Engineering ε-polylysine-based photodynamic therapy agents with oxygen carrying and membrane-targeting capabilities for enhanced therapy under hypoxic conditions

Lianjun Mou [a], Xiaoyan Lou [b], Chang Liu [b], Qian Yuan [b], Qingmeng Zhang [c, *], Xilei Xie [b], Xiaoyun Jiao [b, e], Heng Liu [d, *], Jian Zhang [b, e, *]

[a] Department of Pathology, Hunan University of Medicine General Hospital, Huaihua 418000, China

[b] College of Chemistry, Chemical Engineering and Materials Science, Shandong Normal University, Jinan 250014, China

[c] Department of Orthopaedics, Qilu Hospital of Shandong University, Jinan 250012, China

[d] Key Laboratory of Reproductive Health Diseases Research and Translation of Ministry of Education, The First Affiliated Hospital, Hainan Medical University, Haikou 571199, China

[e] State Key Laboratory of Molecular Engineering of Polymers, Fudan University, Shanghai 200438, China.

E-mail addresses: zhangqingmeng@email.sdu.edu.cn; liuheng11b@muhn.edu.cn; zhangjian12b@mails.ucas.ac.cn

Materials and methods

Materials and instrumentation

The absorption spectra were measured on a UV-1700 spectrophotometer (Shimadzu, Japan). The ^{1}H NMR and ^{19}F NMR spectra were acquired on a nuclear magnetic resonance spectrometer (400 MHz, Bruker Co., Ltd., Germany). The δ value represents the shift of the spectrum relative to TMS ($(CH_3)_4Si$ = 0.00 ppm), expressed in ppm. Confocal imaging data were obtained on TCS SP8 confocal laser scanning microscope (Leica Co., Ltd., Germany). The data of MTT experiment was measured with a microplate reader (TRITURUS). The light sources included 645 nm LED source (CCS Int. 210 mW/cm^2).

5,10,15,20-tetra (4-carboxyl phenyl) porphyrin, N-Hydroxysuccinimide, Perfluorohexanoic acid, N-(3-dimethylaminopropyL)-N'-ethylcarbodiimide hydrochloride, D-biotin and 9,10-anthracenediyl-bis(methylene) dimalonic acid were purchased from Energy Chemical. 2′,7′-Dichlorodihydrofluorescein Diacetate was purchased from BOSTER Biological Technology Co., LTD. ε-Polylysine (MV: 2000-5000) was purchased from Aladdin Co., LTD. Regenerated cellulose dialysis bags (SP132640-1 m, 45.0 mm, 6.4 ML/CM, MWCO: 1.0 KDa) and clips (6.0 cm, 8.0 cm) were purchased from Shanghai Yuanye Co., LTD. The Calcein-AM/ Propidium Iodide (PI) double staining kit and the YF488-Annexin V/ Propidium Iodide (PI) apoptosis detection kit were purchased from UE. Common solvents were purchased from Sinopharm Chemical Reagent Co., Ltd. All reagents and drugs can be used directly without further purification.

Synthesis of Compound PLY-1

A mixture of 5,10,15,20-tetra (4-carboxyl phenyl) porphyrin (H_2TCPP, 0.05 mmol, 40.0 mg), N-(3-dimethylaminopropyL)-N'-ethylcarbodiimide hydrochloride (EDC, 0.40 mmol, 76.7 mg), and N-hydroxysuccinimide (NHS, 0.40 mmol, 46.1 mg) were dissolved in DMF, the mixture was stirred at room temperature for 4 h in the dark. Subsequently, ε-polylysine (2.00 mmol, 316.0 mg) dissolved in DMF was added, and the mixture was stirred at 60 °C under argon protection for 4 h, and then the mixture was moved to room temperature for 20 h. At the same time, Perfluorohexanoic acid (PF-HA, 0.20 mmol, 72.8 mg) and D-biotin (D-Bio, 0.01 mmol, 2.5 mg) were respectively activated by reacting with EDC and NHS in argon at 60 °C for 4 h, and stirring at room temperature for 20 h, then added to the above solution. After completion of the reaction, the reaction solution was dialyzed with a regenerated cellulose dialysis bag (MWCO: 1.0 KDa) (H_2O: EtOH = 3:7) for 72 h, and the dialysate was changed every 4 h. Finally, the PLY-1 was obtained by lyophilization.

Calculation of the grafted number of D-biotin and H_2TCPP in PLY-1

For quantitative analysis, the molecular weight of poly-L-lysine (PLL) was set at 3500 (within the reported range of 2000-5000 of commercial PLL, with an average value of 3500). Considering that the molecular weight of a lysine residue was 128, the degree of polymerization of PLL was calculated to be approximately 27. In the ^{1}H NMR spectrum of PLY-1 (Figure S2), the characteristic resonance peaks at 3.74 ppm (methylene protons of PLL), 2.33 ppm (methylene protons adjacent to the carboxyl group), and −3.04 ppm (shielded protons of the porphyrin derivative) were selected as reference signals for integration. After calibration of the corresponding integral areas, the average grafting numbers of biotin and the photosensitizer molecules in PLY-1 were determined to be 0.63 per PLL chain. This statistically averaged and reasonable result indicated that the conjugation reaction was successfully achieved, suggesting that approximately one-third of the polymer backbones carried a single grafted moiety. Furthermore, because PF-HA contained no hydrogen atoms, its degree of grafting could not be quantified using this NMR-based method. The substitute ratio in PLY-2 and PLY-3 was also determined with this method.

$^1O_2$ generation

The ability of $^1O_2$ generation of PLY-1 was evaluated by 9,10-anthracenediyl-bis(methylene) dimalonic acid (ABDA). ABDA can be oxidized by $^1O_2$, resulting in a decrease in its own absorption. The mixture of PLY-1 (1.0 mg/mL) and ABDA (1.0 mM) was irradiated with a 645 nm LED (210 mW/cm^2) red light in an aqueous solution. The UV-Vis spectra of ABDA photooxidation were recorded every 5 min for a total of 30 min. The effects of ε-polylysine, D-biotin and perfluorohexanoic acid on the absorption spectrum of ABDA were determined by the same method.

PLY-1 +O_2

PLY-1 solution was placed in liquid nitrogen at -78 °C. After the solution was frozen, it was vacuumized (20 min) to remove the dissolved oxygen in the solution. After thawing naturally at room temperature, the solution was fed with high-purity oxygen for oxygenation (bubbling for 1 min).

Cell incubation

HeLa cells were seeded in DMEM medium containing 10% FBS, 1% penicillin and 1% streptomycin in confocal cell culture dishes and cultured in an incubator (37 °C, 5%CO_2/95% air) for 24 h.

Cell uptake experiment

The uptake of PLY-1 by cancer cells was studied in HeLa cells. The PLY-1 (10.0 μg/mL) and cells were co-incubated for 0 min, 10 min, 20 min, 30 min and 40 min, respectively. The real-time imaging was performed to monitor cell uptake using a confocal fluorescence microscope (λ_{ex} = 405 nm, collection channel: 600-750 nm, scale: 25 μm).

In vitro cytotoxicity assays

HeLa cells were seeded in eight identical 96-well cell culture plates (the volume is 200 μL/well), followed by incubating in an incubator (37°C, 5% CO_2/95% air) for 24 hours. The eight 96-well plates were then treated in different ways (four of them were placed in sealed AnaeroPouch hypoxic bag, which reached hypoxia (O_2 concentration <1.0%) within 1 h, and the remaining four were left untreated) and incubated for another 12 h. Then, the cells in different groups were incubated with different concentrations (0, 0.5, 1.5, 2.0, 2.5, 5.0, 10.0 and 13.0 μg/mL) of PLY-1 or PLY-1/O_2 for 30 min in the dark, and the cells in each group were treated differently. For normoxia conditions: group1, PLY-1 was added and incubated for another 12 h; group 2, after addition of PLY-1 and irradiation (5 min·well^{-1}) with 645 nm LED (210 mW/cm^2) red light, the culture medium was aspirated and washed with PBS, and the cells were further cultured for 12 h; group 3, PLY-1/O_2 was added and incubated for another 12 h; group 4, after addition of PLY-1/O_2 and irradiation (5 min·well^{-1}) with 645 nm LED (210 mW/cm^2) red light, the culture medium was aspirated and cells were washed with PBS and then cultured for 12 h. For hypoxic conditions: group 1, PLY-1 was added and incubated in AnaeroPouch bags for 12 h; group 2, after addition of PLY-1 and irradiation (5 min·well^{-1}) with 645 nm LED (210 mW/cm^2) red light, the culture medium was aspirated and washed with PBS, and then the cells were cultured in AnaeroPouch bag for 12 h; group 3, PLY-1/O_2 was added and incubated in AnaeroPouch bag for another 12 h; group 4, after addition of PLY-1/O_2 and irradiation (5 min·well^{-1}) with 645 nm LED (210 mW/cm^2) red light, the culture medium was aspirated and washed with PBS, and then the cells were cultured in AnaeroPouch bag for 12 h. Subsequently, 200 μL MTT solution (5.0 mg·mL^{-1}) in PBS was added to each well. After 4 h, the remaining MTT solution was removed and 200 μL DMSO was added to each well. The TRITURUS microplate reader was used to measure the absorbance of the solution at 490 nm.

Intracellular $^1O_2$ detection

The $^1O_2$ indicator DCFH-DA was used to detect $^1O_2$ produced by PLY-1 in HeLa cells. In brief, HeLa cells were seeded in confocal dishes and then incubated with PLY-1 (10.0 μg/mL) for 30 min, and washed three times with PBS, incubated with

20.0 μM DCFH-DA for 30 min, then washed with PBS; After irradiation of the cells with 645 nm LED (210 mW/cm^2) red light for 5 min, confocal fluorescence imaging was performed to detect the level of singlet oxygen (λ_{ex} = 488 nm, collection channel: 500-580 nm, scale bar: 100 μm).

Live and dead cell staining

Calcein-AM and PI cellular viability kit was used to stain HeLa cells for confocal imaging. In brief, HeLa cells were seeded in confocal dishes and then incubated with different subsequent treatments: group 1, control; group 2, irradiated with 645 nm LED (210 mW/cm^2) red light for 5 min; group 3, incubated with PLY-1 (10.0 μg/mL) for 30 min; group 4, incubated with PLY-1 (10.0 μg/mL) for 30 min , then irradiated with 645 nm LED (210 mW/cm^2) red light for 5 min; group 5, incubated with PLY-1 + O_2 (10.0 μg/mL) for 30 min; group 6, incubated with PLY-1 + O_2 (10.0 μg/mL) for 30 min, then irradiated with 645 nm LED (210 mW/cm^2) red light for 5 min. After treatment, cells were stained with 2.0 μM Calcein-AM and 4.5 μM PI cellular viability kit according the manual. The live-dead cell imaging was observed by confocal microscope (Calcein-AM excitation source: 488 nm, collection channel: 500-550 nm, PI excitation source: 543 nm, collection channel: 580-650 nm, scale: 250μm).

Apoptosis test

YF488-Annexin V/propidium iodide (PI) Apoptosis Detection Kit was used to perform double-stain imaging of photoinduced cell death of HeLa cells. In brief, HeLa cells were seeded in confocal dishes and then incubated with different subsequent treatments: group 1, control; group 2, irradiated with 645 nm LED (210 mW/cm^2) red light for 5 min; group 3, incubated with PLY-1 (10.0 μg/mL) for 30 min; group 4, incubated with PLY-1 (10.0 μg/mL) for 30 min , then irradiated with 645 nm LED (210 mW/cm^2) red light for 5 min; group 5, incubated with PLY-1 + O_2 (10.0 μg/mL) for 30 min; group 6, incubated with PLY-1 + O_2 (10.0 μg/mL) for 30 min, then irradiated with 645 nm LED (210 mW/cm^2) red light for 5 min. After treatment, cells were stained with YF488-Annexin V/PI Apoptosis Detection Kit according the manual. The apoptosis imaging was observed by confocal microscope (YF488 excitation light source: 488 nm, collection channel: 490-560 nm; PI excitation light source: 561 nm, collection channel: 600-680 nm, scale bar: 250 μm). In addition, the fluorescence intensity of the above treated cells was quantitatively analyzed by flow cytometry.

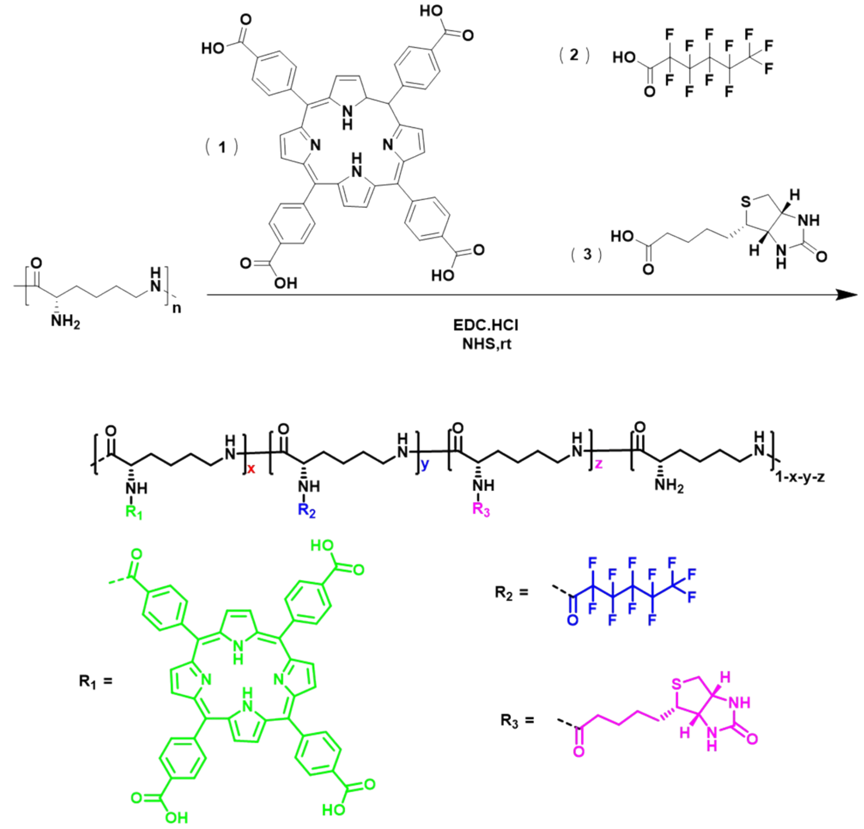

Figure S1 Synthetic route of PLY-1/2/3.

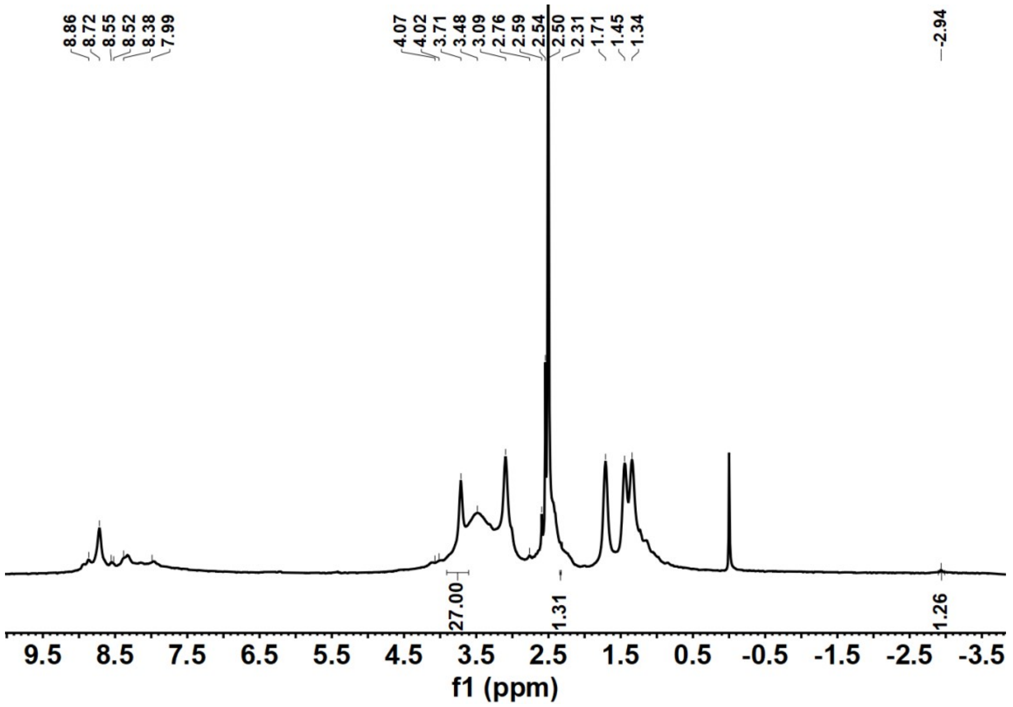

Figure S2 ^{1}H NMR spectrum of PLY-1 in deuterated DMSO.

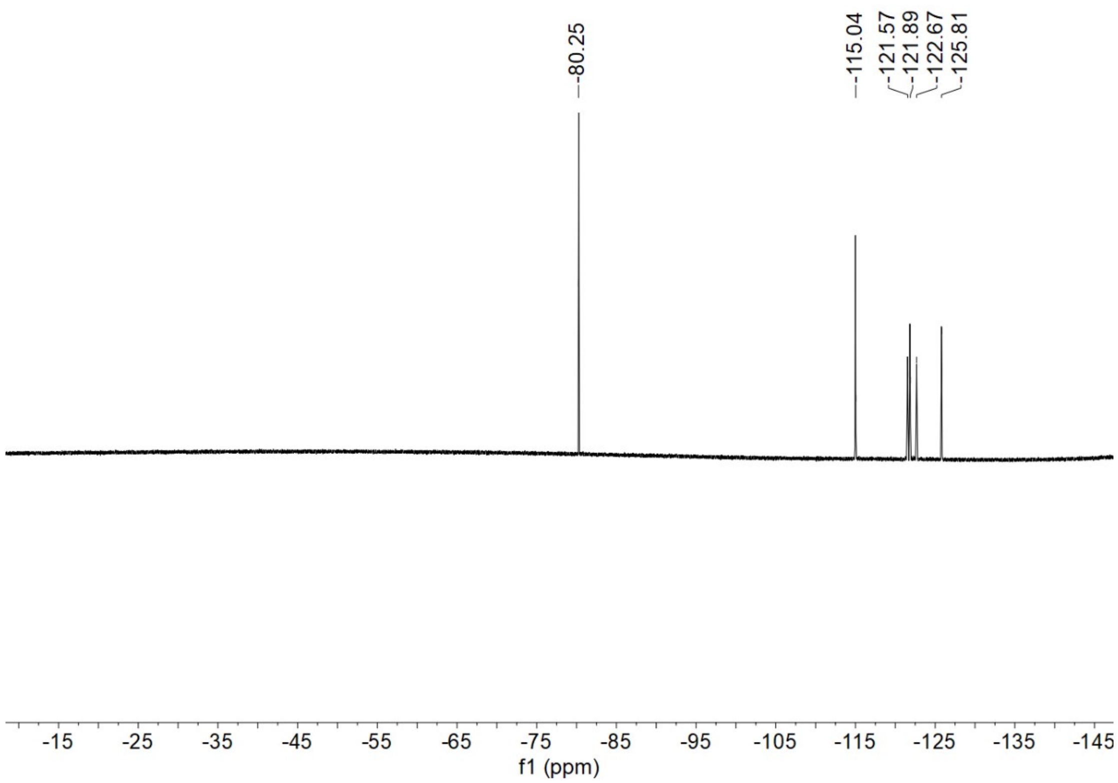

Figure S3 ^{19}F NMR spectrum of PLY-1 in deuterated DMSO.

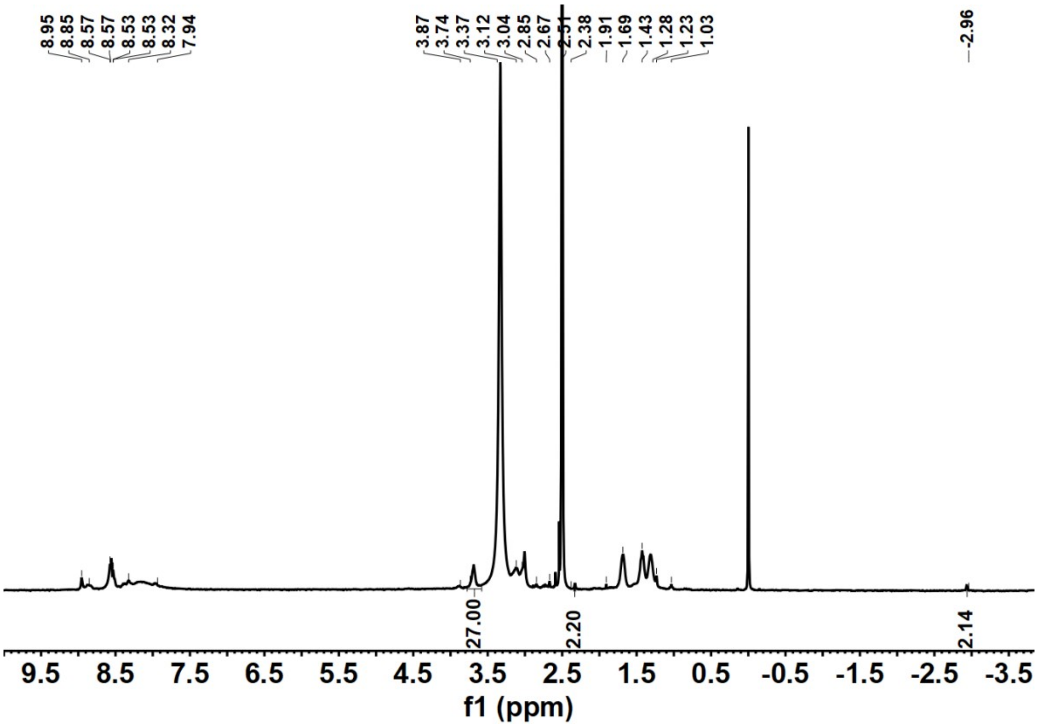

Figure S4 ^{1}H NMR spectrum of PLY-2 in deuterated DMSO.

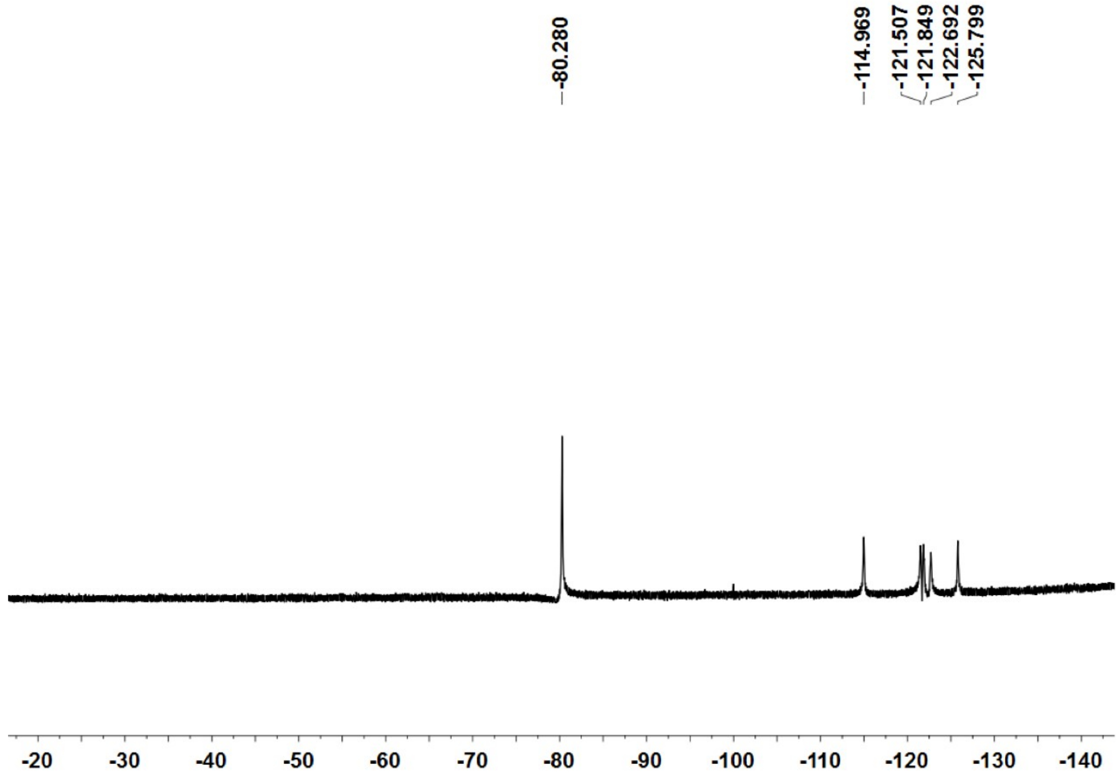

Figure S5 ^{19}F NMR spectrum of PLY-2 in deuterated DMSO.

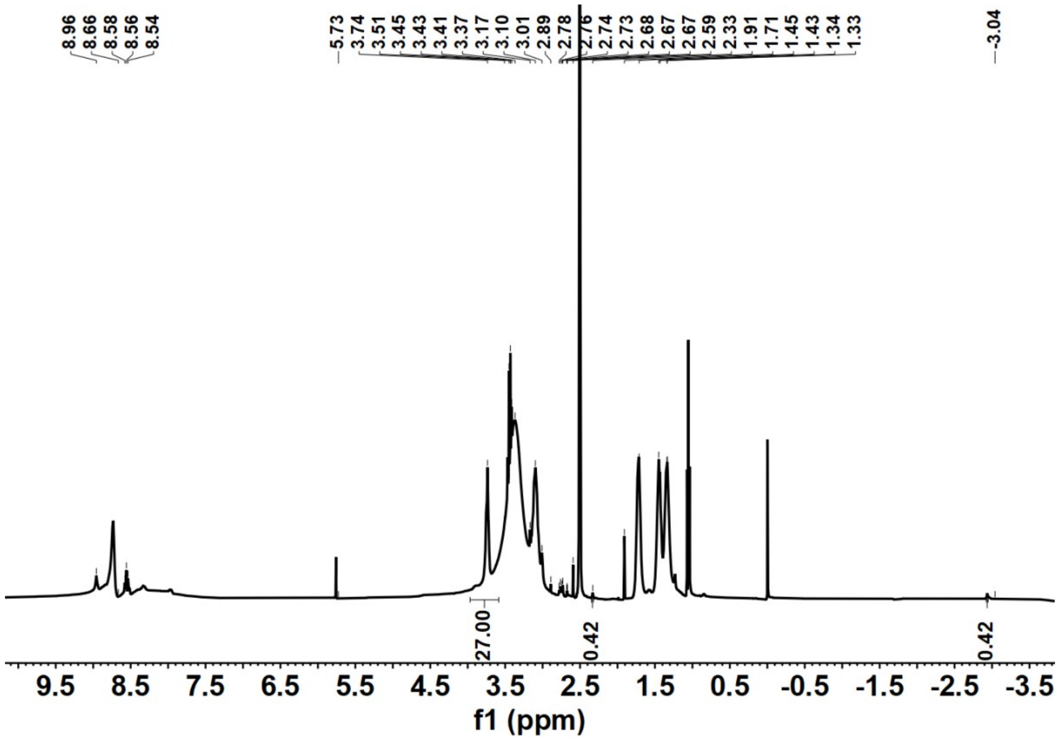

Figure S6 ^{1}H NMR spectrum of PLY-3 in deuterated DMSO.

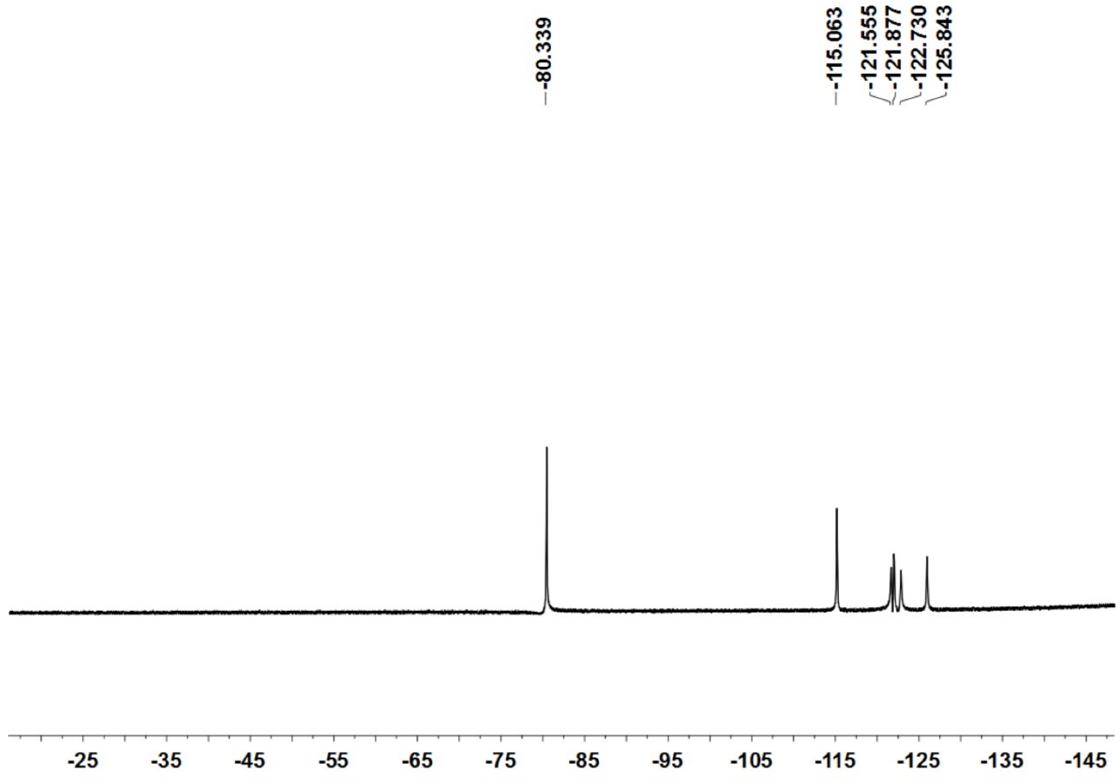

Figure S7 ^{19}F NMR spectrum of PLY-3 in deuterated DMSO.

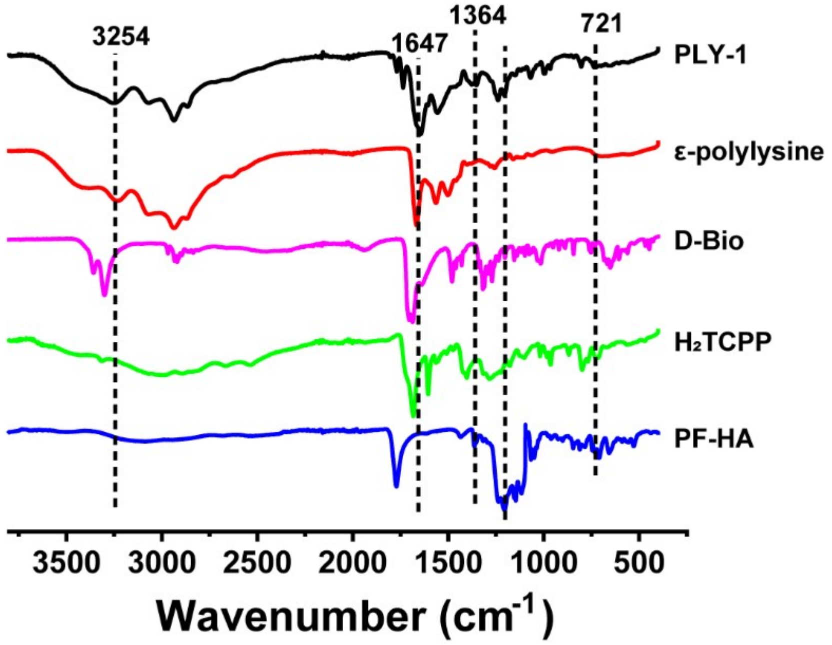

Figure S8 The fourier transform infrared spectroscopy (FTIR) of PLY-1, H_2TCPP, D-Bio, ε-polylysine and PF-HA.

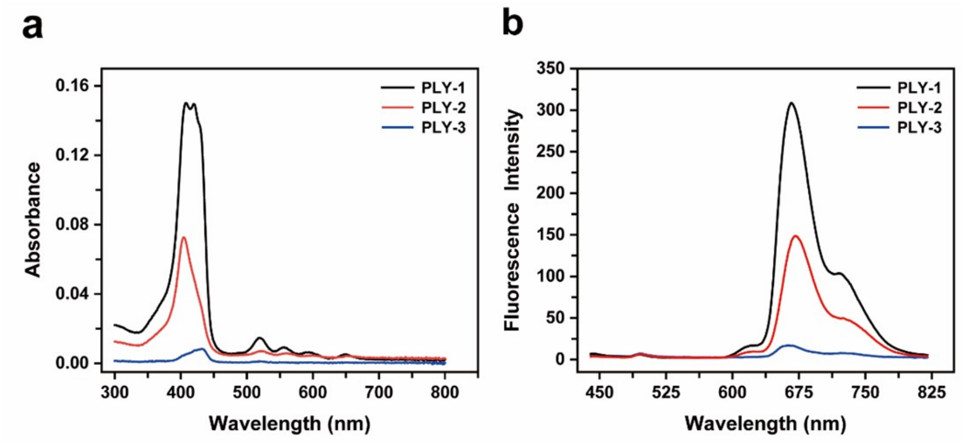

Figure S9 The normalized absorption and emission spectra of PLY-1/2/3 (10.0 μg/mL).

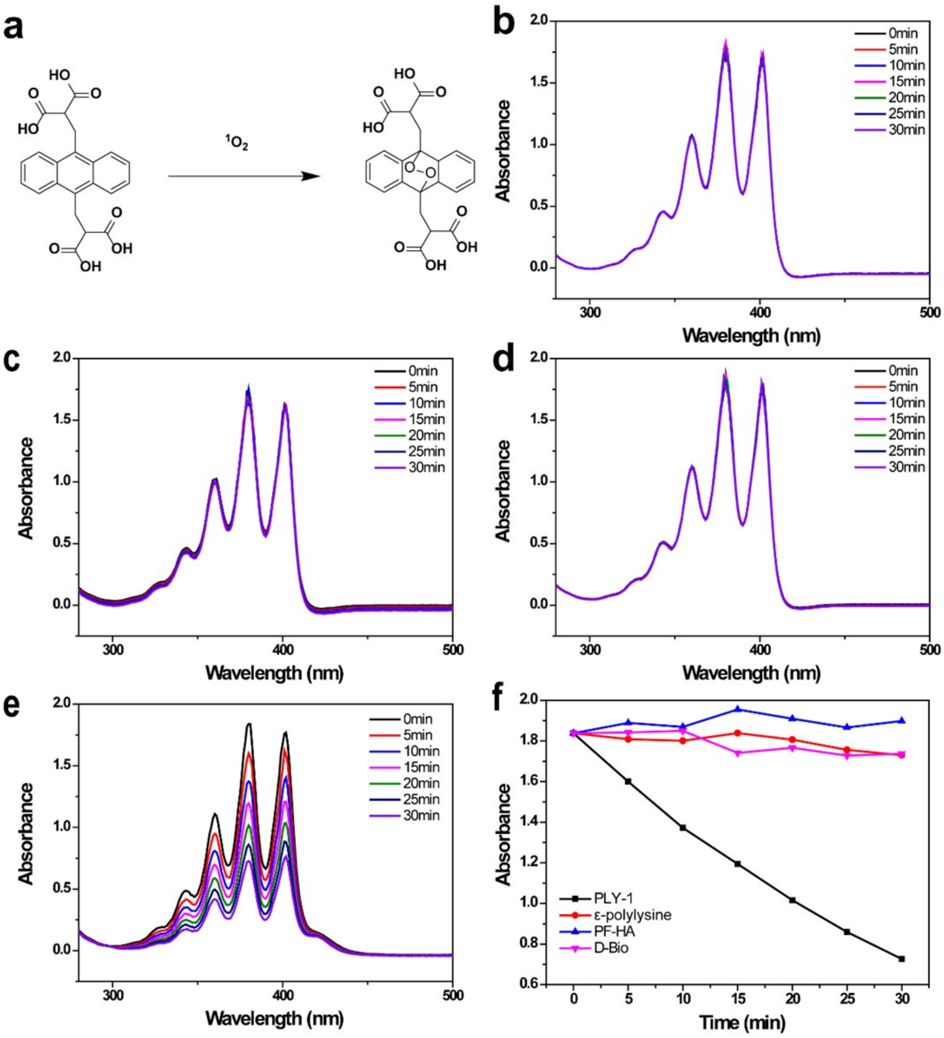

Figure S10 (a) Mechanism diagram of the reaction between ABDA and $^1O_2$; (b) The evolution of UV-Vis absorption spectra of ABDA in PF-HA solution irradiated for different durations with light irradiation (645 nm, 210 mW/cm^{-2}); (c) The evolution of

UV-Vis absorption spectra of ABDA in D-Bio solution irradiated for different durations with light irradiation (645 nm, 210 mW/cm^{-2}); (d) The evolution of UV-Vis absorption spectra of ABDA in ε-polylysine solution irradiated for different durations with light irradiation (645 nm, 210mW/cm^{-2}); (e) The evolution of UV-Vis absorption spectra of ABDA in PLY-1 solution irradiated for different durations with light irradiation (645nm, 210 mW/cm^{-2}); (f) Degradation rates of ABDA by PLY-1, PF-HA , D-Bio and ε-polylisine at 378 nm, respectively.

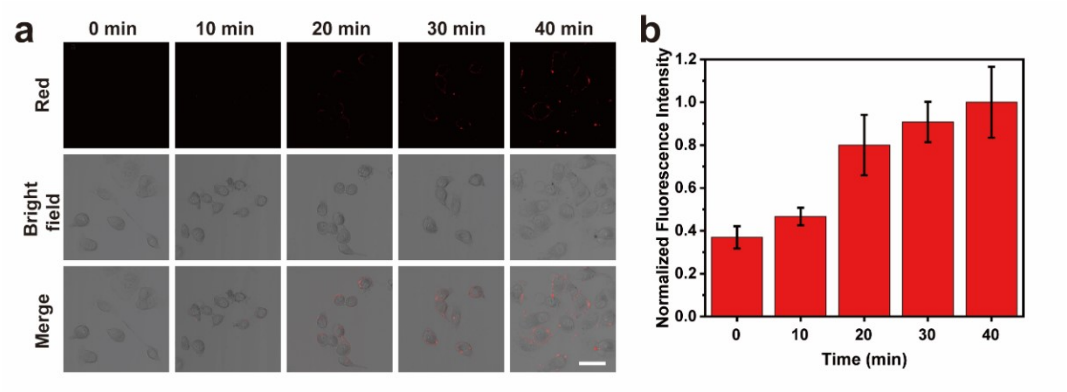

Figure S11 Real-time uptake images and quantification of PLY-1 (10.0 μg/mL) in HeLa cells. Cells were incubating with PLY-1 (10.0 μg/mL), and then confocal fluorescence images were recorded at different time points (0 min, 10 min, 20 min, 30 min and 40 min). (λ_{ex}: 405 nm, collection channel: 600-750 nm, scale: 25 μm).

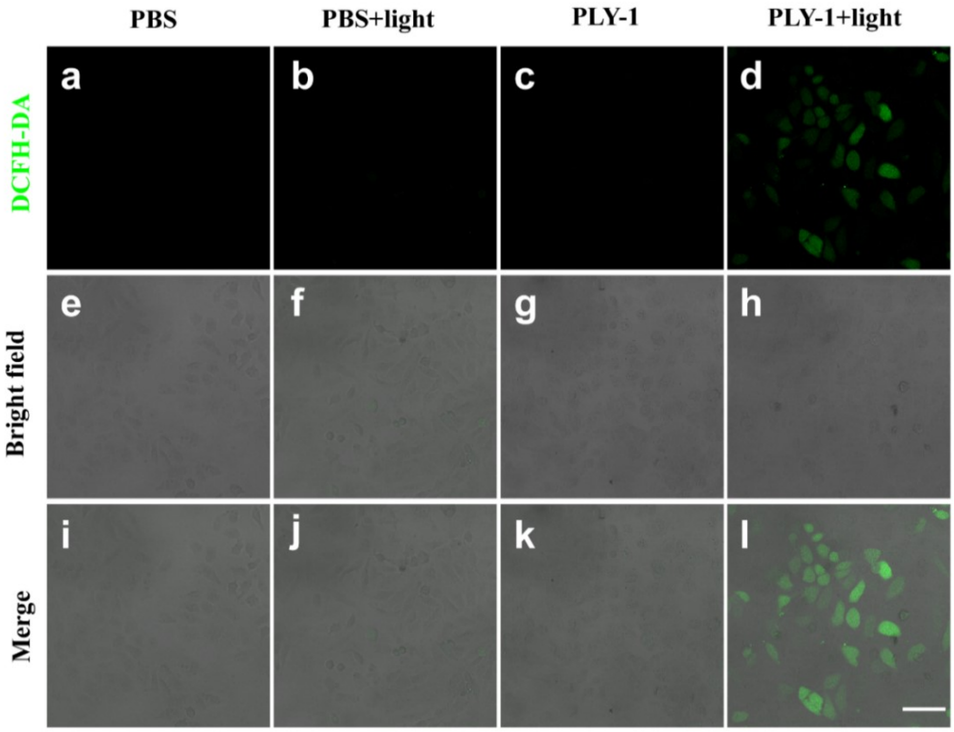

Figure S12 CLSM images of HeLa cells stained with DCFH-DA. The treatments included PBS, PBS + light, PLY-1, PLY-1 + light. (Light: 645 nm, 210 mW/cm^2, 5 min; excitation source: 488 nm, collection channel: 500-580 nm, scale bar: 100 μm).

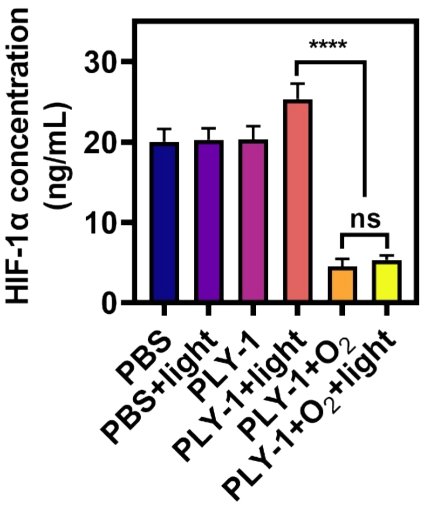

Figure S13 Quantitative analysis of HIF-1α expression in HeLa cells under hypoxic conditions. Data were presented as mean ± SD (n = 3, one-way ANOVA, ****p < 0.0001).

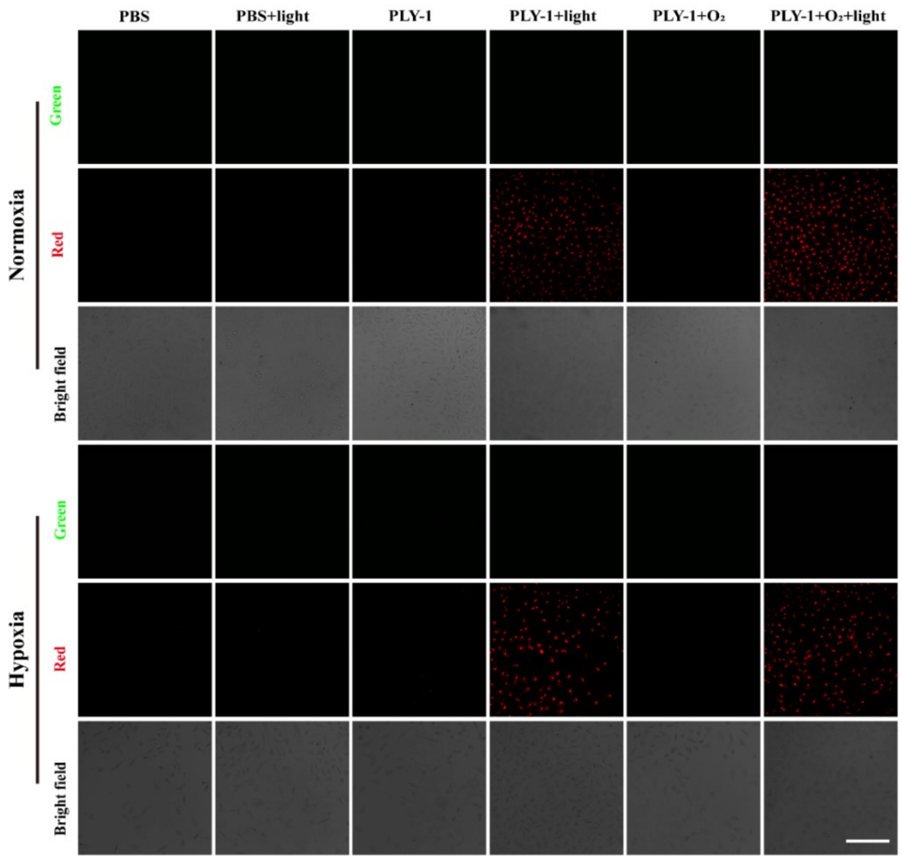

Figure S14 CLSM images of Hela cells incubated with YF488-Annexin V/PI after different treatments in normoxia and hypoxia respectively. The treatments included PBS, PBS + light, PLY-1, PLY-1 + light, PLY-1 + O_2 and PLY-1 + O_2 + light groups (light: 645 nm, 210 mW/cm^2, 5 min; YF488 excitation source: 488 nm, collection channel: 490-560 nm, PI excitation source: 561 nm, collection channel: 600-680 nm, scale: 250 μm).
